# Health service use by same-sex attracted Australian women for alcohol and mental health issues: a cross-sectional study

**DOI:** 10.3399/bjgpopen18X101565

**Published:** 2018-05-16

**Authors:** Ruth McNair, Amy Pennay, Tonda L Hughes, Scarlet Love, Jodie Valpied, Dan I Lubman

**Affiliations:** 1 Honorary Associate Professor, Department of General Practice, University of Melbourne, Carlton, Australia; 2 Research Fellow, School of Psychology and Public Health, Centre for Alcohol Policy Research, La Trobe University, Melbourne, Australia; 3 Professor of Nursing in Psychiatry and Director of Global Health Research, Department of Psychiatry, School of Nursing, Columbia University, New York, US; 4 Resident Medical Officer, Royal Melbourne Hospital, Parkville, Australia; 5 Honorary Lecturer, Department of General Practice, University of Melbourne, Carlton, Australia; 6 Director, Turning Point, Eastern Health Clinical School, Monash University, Melbourne, Australia; 7 Professor of Addiction Studies and Services, Monash University, Melbourne, Australia

**Keywords:** same-sex attracted women, general practitioner, health service use, mental health, alcohol

## Abstract

**Background:**

Same-sex attracted women (SSAW) have higher rates of alcohol and mental health problems than heterosexual women, but utilisation of and satisfaction with treatment is limited.

**Aim:**

This study investigated the influences on health service use for alcohol and mental health problems among SSAW.

**Design & setting:**

The Gelberg-Andersen behavioural model of health service utilisation was used to generate outcome variables.

**Method:**

A convenience sample of 521 community-connected Australian SSAW completed an online survey. Health service use according to sexual identity was compared using χ^2^ analysis. Binary logistic regression examined associations between the independent variables with treatment utilisation.

**Results:**

Reports of alcohol treatment were very low. Only 41.1% of participants with service need had utilised mental health and alcohol treatment. Bisexual women (adjusted odds ratio [AOR] = 2.76) and those with ‘other’ identities (AOR = 2.38) were more likely to use services than lesbian women. Enablers to service use were having a regular GP (AOR = 3.02); disclosure of sexuality to the GP (AOR = 2.42); lesbian, gay, bisexual and transgender (LGBT) community-connectedness (AOR = 1.11); and intimate partner violence ([IPV] AOR = 2.51). Social support was associated with a reduction in treatment use (AOR = 0.97). Significant access barriers included not feeling ready for help, and previous negative experiences related to sexual identity.

**Conclusion:**

Disclosing sexual identity to a regular, trusted GP correlated with improved utilisation of alcohol and mental health treatment for SSAW. The benefits of seeking help for alcohol use, and of accessing LGBT-inclusive GPs to do so, should be promoted to SSAW.

## How this fits in

This study improves understanding of the barriers and enablers of healthcare utilisation for SSAW, particularly in relation to treatment for alcohol problems. GPs are pivotal to improving health care for SSAW through providing a trustworthy environment in which sensitive issues can be disclosed.

## Introduction

SSAW have significantly higher rates of problematic drinking than heterosexual women.^1–4^ Bisexual and ‘mainly heterosexual’ women demonstrate even higher risk than lesbian women. 'Mainly heterosexual' signifies people who are same-sex attracted, but feel somewhere between bisexual and heterosexual in terms of their sexual identity.^5^ Mainly heterosexual young Australian women are significantly more likely than heterosexual women to report drinking >15 standard drinks per week, and mainly heterosexual and bisexual women were more likely to report binge drinking.^6 ^Depression and anxiety are also significantly higher for SSAW, particularly bisexual and mainly heterosexual women.^7–9^ Problematic drinking is defined as drinking beyond levels set by national guidelines, and is generally defined by the research team or clinicians in the literature, rather than by the research participant. Problematic drinking is associated with stress,^10^ depression,^11^ childhood trauma, and sexuality-related discrimination.^12,13^ Sexual re-victimisation is also a strong predictor of problematic drinking, particularly for lesbian and mainly heterosexual women,^14^ and alcohol use is strongly associated with IPV in lesbian couples.^15^ Gender-diverse and transgender (trans) people may also be same-sex attracted, or attracted to other gender-diverse people (pansexual). Currently, there are no population-based Australian data on drinking patterns among trans and gender-diverse people.

Higher rates of problematic drinking do not correlate well with treatment-seeking for alcohol-related problems among SSAW.^6,16,17^ SSAW are consistently less likely than heterosexual women to use alcohol treatment services, despite reporting more problematic drinking.^3^ Seventy percent of Australian same-sex attracted people aged 18–25 years reported problematic alcohol use, but only 6% reported accessing any form of treatment.^13^ Mental healthcare utilisation can also be problematic. For example, a Canadian study found higher levels of unmet need for mental health care among trans women (2.4 times, *P*<0.01) and bisexual women (1.8 times, *P* = 0.02).^17^ SSAW consistently report unmet needs and low levels of satisfaction with alcohol and mental health treatment.^18–20^

Women as a whole encounter barriers to accessing addiction services, which contributes to their seeking help online,^21^ but there are compounding barriers for SSAW. Lesbian, gay, and bisexual (LGB) people in the US were more likely than heterosexual people to report 'perceptual’ barriers to alcohol treatment, such as believing they should handle the problem alone, or that the problem was not serious enough.^4^ LGB people also face discrimination and assumptions of heterosexuality that lead to poor communication and treatment planning.^22^ Alcohol treatment may be more accessible to SSAW if it is tailored specifically to their needs,^23^ is culturally inclusive, and is 'gay affirmative.’^24^ Factors found to enable mental health care-seeking, that may also influence alcohol treatment, include having a regular GP, community-connectedness,^22^ and disclosure of sexual identity to a supportive GP.^25–27^

There is limited research that describes the relative factors influencing alcohol and mental health treatment-seeking. This study addressed this gap by applying the Gelberg-Andersen Behavioural Model for Vulnerable Populations to examine perceptual, structural, and social factors influencing SSAW’s help-seeking.^28^ The model frames health service use as a function of predisposing, enabling, and need characteristics. Predisposing characteristics are those that 'exist prior to the perception of illness', and include age, gender, education, race, and victimisation. Enabling characteristics, such as income or social support, facilitate service access once illness has been perceived, or inhibit service access if lacking. Need characteristics include the type and severity of relevant health conditions.^29^ This study focused on characteristics specific to SSAW. The Gelberg-Andersen model has previously been used with SSAW in relation to treatment utilisation and alcohol and mental health disorders.^30^ Based on the literature, it was hypothesised that specific influences would vary according to predisposing characteristics (such as sexual identity and IPV history); enabling or inhibiting characteristics (for example, having a regular GP, level of social support, and discrimination); and need characteristics (such as depression, problematic drinking, or suicide attempts).

## Method

### Study design

Data from the Alcohol and Lesbian/bisexual women: Insights into Culture and Emotions (ALICE) project were collected online from adult SSAW living in Australia. Participants were recruited using social media, community listings and events, and snowballing, and all responders were included in the study. The primary research question was 'what are the barriers and enablers to the use of health services for mental health and alcohol-related problems among SSAW?'.

### Measures

The primary outcome variable was treatment utilisation. Other measures are grouped within each of three categories suggested by the Gelberg-Andersen model.

#### Treatment utilisation

Participants were asked if they had 'attended any of these services' (for example, the GP) 'for help with mental health and/or emotional wellbeing', and 'for help with alcohol use' within the previous 12 months (Table 1). For those that answered affirmatively, frequency of attendance was recorded.

**Table 1.** Twelve-month treatment utilisation for mental health and alcohol-related problems by service provider

*N* = 521	Treatment for mental health problems in past 12 months, *n* (%)			Treatment for alcohol-related problems in past 12 months, *n* (%)		
	**Once**	**>Once**	**Total**	**Once**	**>Once**	**Total**
**Type of health service**						
Counsellor, psychologist, or psychiatrist	18 (3.5)	188 (36.1)	206 (39.5)	4 (0.8)	33 (6.3)	37 (7.1)
GP	61 (11.7)	135 (25.9)	196 (37.6)	12 (2.3)	9 (1.7)	21 (4.0)
Specialist doctor	20 (3.8)	49 (9.4)	69 (13.2)	5 (1.0)	8 (1.5)	13 (2.5)
Nurse	18 (3.5)	17 (3.3)	35 (6.7)	8 (1.5)	4 (0.8)	12 (2.3)
Social worker	15 (2.9)	16 (3.1)	31 (6.0)	2 (0.4)	4 (0.8)	6 (1.2)
General telephone counselling	14 (2.7)	22 (4.2)	36 (6.9)	2 (0.4)	4 (0.8)	6(1.2)
Emergency department	16 (3.1)	8 (1.5)	24 (4.6)	6 (1.2)	3 (0.6)	9 (1.7)
Drug or alcohol-specific telephone counselling	–	–	–	4 (0.8)	4 (0.8)	8 (1.5)
Drug or alcohol service in person	–	–	–	1 (0.2)	6 (1.2)	7 (1.3)
Self-help group	6 (1.2)	13 (2.5)	19 (3.6)	4 (0.8)	14 (2.7)	18 (3.5)
Police	6 (1.2)	5 (1.0)	11 (2.1)	3 (0.6)	2 (0.4)	5 (1.0)
Other	4 (0.8)	9 (1.7)	13 (2.5)	1 (0.2)	3 (0.6)	4 (0.8)

#### Predisposing variables (Table 2)

**Table 2.** Sample demographics by 12-month treatment utilisation for mental health and alcohol-related problems

	Any treatment for mental health or alcohol-related problems within 12 months (*n *= 271), *n* (%)	No treatment for mental health or alcohol-related problems within 12 months (*n *= 250), *n* (%)	Total (*n *= 521)
**Mean age (SD)**	33.35 (11.6)	34.43 (13.7)	33.87 (12.7)
**Gender**			
Female	242 (89.3)	237 (94.8)	479 (91.9)
Trans (identifying as female)	6 (2.2)	2 (0.8)	8 (1.5)
Trans (identifying as male)	1 (0.4)	0 (0.0)	1 (0.2)
Genderfluid or genderqueer	7 (2.6)	5 (2.0)	12 (2.3)
Other	15 (5.5)	6 (2.4)	21 (4.0)
**Sexual identity**			
Lesbian	143 (52.8)	154 (61.6)	297 (57.0)
Bisexual	54 (19.9)	35 (14.0)	89 (17.1)
Queer/pansexual	56 (20.7)	43 (17.2)	99 (19.0)
Other	18 (6.6)	18 (7.2)	36 (6.9)
**In a relationship**	136 (50.2)	162 (64.8)	298 (57.2)
**Has children**	50 (18.5)	50 (20.0)	100 (19.2)
**Income ≤AUS $40 000 per annum**	89 (32.8)	50 (20.0)	139 (26.7)
**Unemployed**	39 (14.4)	23 (14.3)	62 (11.9)
**Left high school before completion**	16 (5.9)	15 (6.0)	31 (6.0)
**Born outside Australia**	42 (15.5)	45 (18.0)	87 (16.7)
**Aboriginal or Torres Strait Islander**	6 (2.2)	7 (2.8)	13 (2.5)
**Lives in rural locality**	29 (10.7)	32 (12.8)	61 (11.7)

SD = standard deviation. Trans = transgender.

Participants were asked their sexual identity: 'lesbian/gay', 'queer', 'bisexual', 'heterosexual/straight', 'not sure or undecided', or 'I prefer to refer to myself as…'. These were recoded into lesbian/gay, bisexual, queer/pansexual, and ‘other’. Queer identity tends to be more fluid and is distinct from lesbian or bisexual. Any heterosexual participants were included in the ‘other’ category. Participants were also asked their gender identity (for example, trans, genderqueer, or other). IPV was measured using three questions asking whether participants had ever been emotionally, sexually, or physically abused by a partner (in the past 12 months and before the past 12 months). Any abuse was regarded as IPV.

#### Enabling and inhibiting characteristics (Table 3)

**Table 3.** Binary logistic regression model of influences on past-year treatment utilisation for mental health or alcohol-related problems

			Multivariate model					
			**Unadjusted**			**Adjusted^a^**		
	**Past-year treatment utilisation,** ***n* (%)**	**No past-year treatment utilisation,** ***n* (%)**	**Wald**	***P***	**OR (95% CI)**	**Wald**	***P***	**OR (95% CI)**
								
**Level of social support, mean score (SD)**	50.3 (17.3)	58.2 (17.4)	21.220	<0.0001	0.97 (0.96 to 0.98)	15.69	<0.0001	0.97 (0.96 to 0.99)
**Level of LGBT community-connectedness, mean score (SD)**	3.8 (2.9)	3.1 (2.6)	5.452	0.020	1.10 (1.02 to 1.18)	6.67	0.010	1.11 (1.03 to 1.20)
**Has a regular GP**	217 (57.6)	160 (42.4)	17.10	<0.0001	2.79 (1.72 to 4.55)	19.08	<0.0001	3.02 (1.84 to 4.96)
**Has disclosed sexuality to GP**	215 (57.2)	161 (42.8)	9.16	0.002	2.16 (1.31 to 3.57)	11.28	0.001	2.42 (1.45 to 4.06)
**Has experience or fear of discrimination within health services (ever)**	48 (56.5)	43 (43.5)	0.28	0.600	1.16 (0.68 to 1.98)	0.02	0.900	1.04 (0.60 to 1.80)
**Sexual identity**								
Lesbian	143 (48.1)	154 (51.9)	14.74	0.002		12.55	0.006	
Bisexual	54 (60.7)	35 (39.3)	12.37	<0.0001	2.92 (1.60 to 5.30)	10.64	0.001	2.76 (1.50 to 5.08)
Queer	56 (56.6)	43 (43.4)	3.10	0.080	1.67 (0.94 to 2.97)	1.72	0.190	1.47 (0.83 to 2.63)
Other	18 (50.0)	18 (50.0)	4.13	0.040	2.38 (1.03 to 5.47)	3.98	0.046	2.38 (1.02 to 5.57)
**Low income (<AUS $40 000, Yes/No)**	89 (64.0)	50 (36.0)	–	–	–	1.02	0.310	1.28 (0.79 to 2.09)
**Service need**	185 (58.9)	129 (41.1)	–	–	–	6.93	0.008	1.84 (1.17 to 2.90)
**IPV within 12 months**	112 (66.3)	57 (33.7)	17.87	<0.0001	2.64 (1.68 to 4.13)	15.93	<0.0001	2.51 (1.60 to 3.96)
**Model summary**			χ^2^	*P*		χ^2^	*P*	
			88.08	0.000		96.36	0.000	

^a^Sample size = 452, missing data = 69 (cases were excluded if data was missing for at least one variable).

CI = confidence interval. IPV = intimate partner violence. OR = odds ratio. SD = standard deviation.

The 19-item social support scale was used (score range = 0–76), with higher scores indicating greater social support (alpha = 0.97).^31^ An LGBT community-connectedness scale was created (not yet validated) using the following three items:^1^

1. 'In how many LGBT community organisations are you a member?';^2^
2. 'How important is being a member of LGBT community organisations to you?' (possible answers: extremely, very, somewhat, a little, and not at all);^3 ^and
3. 'How often do you participate in LGBT community events?' (possible answers: daily, weekly, monthly, annually, and never).

Each item was treated as a 5-point scale (0–4). Responses to the three items were added (range = 0–12), with higher scores indicating greater connection to the LGBT community. Participants were also asked 'Have any of the following ever made you reluctant to seek help or advice about alcohol use?', followed by a list of common barriers drawn from the literature (Table 4). Participants could select 'yes, in the last 12 months', 'yes, more than 12 months ago', 'no, never', or 'other, please describe'. The same question was asked with regard to 'mental health and/or emotional wellbeing.'

**Table 4.** Barriers to treatment utilisation for mental health and alcohol-related problems among those in need of services (based on high CES-D-10, AUDIT score, or suicide attempts within 12 months)

Barriers *(n *= 314, missing data = 14)	Reluctant to seek help or advice for alcohol use or mental health within the previous 12 months due to the stated reason, *n *(%)
Concerns regarding being judged about alcohol use or mental health issues	116 (38.7)
Not feeling ready to seek help or advice	114 (38.0)
Inability to access available services (for example, due to transport issues or cost of services)	85 (28.3)
Previous services used were not helpful	70 (23.3)
Concerns about confidentiality	60 (20.0)
Lack of information about available services	56 (18.7)
Fear of discrimination based on sexual orientation or gender identity	49 (16.3)
Prior experiences of discrimination based on sexual orientation or gender identity	28 (9.3)

AUDIT = Alcohol Use Disorders Identification Test. CES-D-10 = Centre for Epidemiological Studies Depression Scale-10.

#### Need variable

To understand the association between service use and enablers or inhibitors independent of service need, a ‘health service need’ control variable was created, which included participants who were positive for depression, problematic drinking, or suicide attempt within the past 12 months. Depression in the past 4 weeks was measured using the Centre for Epidemiological Studies Depression Scale-10 (CES-D-10);^32^ those participants who scored ≥11 were considered to be depressed. Problematic drinking in the past 12 months was measured using the Alcohol Use Disorders Identification Test (AUDIT); participants scoring ≥8 were deemed to be ‘problematic drinkers’.^33^ Suicidal thoughts and behaviour questions were 'Have you ever seriously thought about committing suicide?' and 'Have you ever attempted suicide?', with responses 'yes, in the last 12 months', 'yes, more than 12 months ago', and 'no, never'. Participants were not asked about self-perceived need for services.

### Analyses

IBM SPSS Statistics (version 22) was used for data analysis. Pearson χ^2 ^tests were used to examine sexual identity differences in study variables. Past 12-month treatment utilisation for mental health and alcohol-related problems were examined separately, using cross-tabulations according to sample demographic characteristics. Frequencies were calculated for the responses ‘once’ and ‘more than once’ in relation to use of each service. To examine barriers to treatment utilisation, frequencies were calculated for participants who were positive for ‘service need’ (*n *= 314).

Binary logistic regression models were tested using ‘treatment utilisation’ as the outcome. The following independent variables were included simultaneously: ‘social support’, ‘having a regular GP’, ‘disclosure of sexuality to GP’, ‘ever experienced or feared discrimination within health services’, ‘sexual orientation’, and ‘IPV’. Correlations were examined among the independent variable to check for multicollinearity. Analyses controlled for service need and low income, as these were found to be correlated with the outcome variable and at least one independent variable at the *P*<0.05 level. Education, age, and parental status were also considered as potential control variables, but none were correlated with the outcome variable, and were therefore not included in further analyses.

## Results

As summarised in Table 2, nearly two-thirds (*n* = 314, 60.3%) of the sample was positive for service need, and 271 (52.0%) had received treatment for mental health or alcohol use in the last year. Of those positive for service need, only 185 (58.9%) had utilised treatment. Of participants who received services, 8 (1.5%) utilised services for alcohol use alone, 205 (39.3%) utilised services for mental health alone, and 58 (11.1%) utilised services for both alcohol and mental health concerns.

Table 1 describes health providers seen for mental health or alcohol use-related concerns. The most utilised types of service providers were counsellor, psychologist, or psychiatrist. Of the 206 participants who sought help from these providers for mental health, 91.3% (*n* = 188) attended more than once in the past year, indicating some level of continuity of care. GPs were the next most frequently used, but only 68.9% (*n* = 135) had seen the GP more than once for mental health issues. In the Australian healthcare system, GPs are the main access point for publicly subsidised counselling or psychiatrist visits, so some of the GP access will have been to seek a referral, rather than for ongoing care for mental health with the GP. Very few of the participants had utilised care for alcohol issues, with only 7.1% (*n* = 37) seeing mental health providers and 4.0% (*n* = 21) seeing GPs. A minority of participants (*n *= 62, 11.9%) reported having attended LGBT-specific services for mental health; and 16 (3.1%) attended LGBT-specific services for alcohol-related problems.

Comparison of service use according to sexual identity shows some differences in relation to provider types. Significant differences were found for having a GP ('lesbian/gay' participants were more likely *P* = 0.001), and the GP knowing sexual identity ('lesbian/gay' participants were more likely *P* = 0.000), and reluctance to use services for mental health ('queer/pansexual' participants were more reluctant *P* = 0.000). There were no differences by sexual identity in reluctance to use services for alcohol use. The use of services at least once in the past 12 months for alcohol use or for mental health also showed no difference by sexual identity. Comparison of LGBT community-connection differed, with bisexual participants much more likely to have never connected (39.3%, *n* = 34) than lesbian (23.2%, *n* = 66) or queer/pansexual (18.2%, *n* = 17; *P* = 0.000) women.

Barriers to seeking help for those with service need are listed in Table 4. The most frequently reported barrier was concern about mental health or alcohol use being judged (38.7%, *n* = 116). The second most frequent barrier was not feeling ready to seek help (38.0%, *n* = 114), and several participants also indicated that they did not feel the need for help. Fear or actual experiences of discrimination was the least reported barrier. While only 16.3% (*n* = 49) selected fear of discrimination, analysis of the ‘other’ responses from participants revealed that the majority were related to negative past healthcare experiences, or not being able to find LGBT-specific services. This indicates that sexual identity was an important barrier even if discrimination in health care per se was not an issue. Experiences of discrimination were common, and significantly differed according to sexual identity. More bisexual (80.9%, *n* = 70) and lesbian (70.7%, *n* = 204) than queer/pansexual (51.5%, *n* = 50, *P* = 0.000) individuals had experienced discrimination in the past year.

Tests to understand associations with treatment use showed similar levels of association individually for mental health and alcohol-related help, so results of the regression models are presented together (Table 3). All independent variables except ‘discrimination within health services ever’ were significantly associated with treatment use (*P*<0.05). The strongest positive associations, in order, were ‘having a regular GP’, ‘bisexual identity’, 'IPV', ‘disclosure of sexuality to GP’, and ‘LGBT community-connectedness’. Greater social support was associated with reduced likelihood of health service use. Bisexual women (AOR = 2.76, *P *= 0.001) and those with an ‘other identity’ (AOR = 2.38, *P *= 0.046) were more likely to utilise health services than lesbian women, even when controlling for health need. The difference in service use between queer and lesbian participants was not statistically significant (*P* = 0.189).

## Discussion

### Summary

This study identified very low levels of service use for alcohol-related problems, despite high levels of risky drinking. The hypotheses that specific influences on help-seeking would include sexual identity, IPV, and GP support were largely supported. In particular, bisexual women were most likely to utilise health care, but less likely to connect with the LGBT community than lesbian women. It was found that barriers to service use varied by sexual identity, and that GP engagement and LGBT community-connection were strong enablers for health service use. The authors did not expect higher levels of social support to be related to reduced service use. It is likely that people with strong social support were less likely to perceive the need for professional support.

### Strengths and limitations

A strength of the study is its description of health services barriers and facilitators to healthcare use, which may be used to develop health promotion initiatives targeted to specific sub-groups of SSAW. Nevertheless, the study had limitations that should be considered. A volunteer sample was used, recruited largely through LGBT social media. SSAW who were more socially isolated or who were not connected with the LGBT community may be under-represented, limiting generalisability of findings. For example, some bisexually active women identify as heterosexual and do not necessarily connect with LGBT social media.^34^ Although collecting data online facilitated the recruitment of a geographically diverse sample, only those with sufficient computer literacy and internet access could participate. The sample may also under-represent some responders in need of health services, as anxiety or physical health were not assessed. Defining problematic drinking based on the AUDIT rather than self-perception also limited the ability to assess whether failing to perceive drinking as a problem was a barrier to help-seeking. Finally, although participants were asked about their gender identity, the number of trans and gender-diverse people was too small to analyse separately, and the focus for this study was sexual identity. Gender diversity is an important area for future research especially because health care is often delayed due to fears or previous experiences of discrimination in this population group.^35^

### Comparison with existing literature

Previous researchers have also found low levels of help-seeking for alcohol-related problems among SSAW. One study using the Gelberg-Andersen model to investigate access to HIV prevention services found that consumption of alcohol was negatively associated with help-seeking.^36^ One possible reason for low levels of alcohol treatment in primary care is limited enquiry about alcohol intake by GPs.^37^ However, primary care is well-placed to provide initial alcohol screening and brief intervention in an affirming environment.^38^ Key barriers to help-seeking for alcohol and mental health in this study were fears of being judged about alcohol use or mental health issues. While the literature suggests fear of sexual identity-based discrimination as a barrier, in the present study, other negative experiences in health care were more problematic. An affirming clinician response to sexual identity disclosure may encourage disclosure of more troubling issues, such as those related to problematic drinking or mental health concerns, but a discriminatory or dismissive response is likely to create more barriers.^25^

Connection to LGBT community was associated with greater treatment utilisation. This is consistent with previous research findings indicating that mental health help-seeking may be less stigmatised and therefore more likely to be encouraged among SSAW compared with heterosexual women.^2,30^ It may be that connection to the LGBT community encourages help-seeking, perhaps by increasing access to information about LGBT-inclusive services. Conversely, social support was negatively associated with health service use in this study. This is counter to the Gelberg-Andersen model that predicts social support as an enabler of health service use.^28,30^ Others have noted that social support reduces the need for mental health services.^39^ Perhaps participants with good social support in this study sought help for their mental health or alcohol-related problems preferentially through social networks rather than health services. Other Australian research has found peer support groups to be commonly used for mental health concerns, although professional help was more likely when mental health problems were more severe.^22^ These findings can be used in targeted health promotion messages that encourage professional help-seeking for alcohol and mental health concerns.

IPV was also strongly associated with mental health and alcohol treatment utilisation. IPV may be at least as common among same-sex as heterosexual couples, although this is an under-researched and under-recognised problem in the LGBT community.^40^ Possible explanations for the association between IPV and help-seeking include the potential compounding effect of IPV on mental health and substance use problems, which increases the need for help.^15^ In addition, SSAW’s disclosure of IPV to a treatment provider often necessitates disclosure of a same-sex relationship. If disclosure of these two potentially stigmatised issues is met with a positive response, SSAW may be more likely to disclose concerns related to mental health and alcohol use. General practice plays a 'critical role’ for victims of IPV, and enablers include positive GP communication skills and time, careful management of disclosure, and sense of safety.^41^ These may be even more important for SSAW.

### Implications for research and practice

General practice has a strong role to play in the health care of SSAW. Having a regular GP was strongly correlated with alcohol and mental health treatment utilisation. Previous findings suggest that having a regular GP is associated with increased healthcare utilisation in general.^42^ In Australia, GPs both provide alcohol-related and mental health care, and enable referral for publicly rebated specialist services,^43^ so having a regular GP should be associated with increased use of services for mental health and alcohol problems. Very few participants utilised services for mental health without seeing a GP. This finding is important in the context of previous research suggesting that SSAW are less likely to have a regular GP compared with heterosexual women, and are less satisfied with the care received.^44^ Similarly, in a large-scale Canadian probability sample, bisexual and lesbian women were much more likely than heterosexual women to have no regular doctor.^45^ Therefore, enabling SSAW to have a regular GP could improve service use for mental health and alcohol concerns.

Disclosure of sexual identity to GPs was significantly associated with the use of health services for alcohol or mental health concerns, a finding that is consistent with previous research.^46–49^ The association between service use and disclosure to one’s GP may have several explanations. It may be that regular visits increase SSAW’s comfort with their GP, as well as opportunities for disclosure.^25^ Further, if disclosure of sexual identity is sensitively received, this may facilitate disclosure of other sensitive or stigmatised issues such as IPV, mental health problems, and substance use. Previous research has found that LGBT people with mental health or substance use concerns prefer to have their sexual orientation acknowledged and integrated into the management process.^50^

Finally, differences in enablers and barriers to treatment-seeking according to sexual identity could be considered when creating more tailored approaches to health promotion for diverse groups of SSAW. For example, bisexual women were less likely to have a regular GP and to be connected to the LGBT community, both of which were positively associated with utilisation of treatment. Bisexual women may benefit from specific health promotion initiatives via mainstream community avenues, as well as via the increasing diversity of online communities.

In conclusion, targeted community education to diverse groups of SSAW is needed to raise awareness and encourage utilisation of health care for problematic alcohol use and mental health concerns, as well as to raise awareness of where to find inclusive primary care. In parallel, GPs need to ensure they are non-judgemental and encourage disclosure of the intersecting issues of sexual identity, alcohol use, IPV, and mental health issues.

## References

[1] Wilsnack SC, Hughes TL, Johnson TP (2008). Drinking and drinking-related problems among heterosexual and sexual minority women. J Stud Alcohol Drugs.

[2] Hughes T (2011). Alcohol-related problems among sexual minority women. Alcohol Treat Q.

[3] McCabe SE, West BT, Hughes TL (2013). Sexual orientation and substance abuse treatment utilization in the United States: results from a national survey. J Subst Abuse Treat.

[4] Allen JL, Mowbray O (2016). Sexual orientation, treatment utilization, and barriers for alcohol related problems: Findings from a nationally representative sample. Drug Alcohol Depend.

[5] McCabe SE, Hughes TL, Bostwick W (2012). Measurement of sexual identity in surveys: implications for substance abuse research. Arch Sex Behav.

[6] Hughes T, Szalacha LA, McNair R (2010). Substance abuse and mental health disparities: comparisons across sexual identity groups in a national sample of young Australian women. Soc Sci Med.

[7] Gilman SE, Cochran SD, Mays VM (2001). Risk of psychiatric disorders among individuals reporting same-sex sexual partners in the national comorbidity survey. Am J Public Health.

[8] Cochran SD, Mays VM, Sullivan JG (2003). Prevalence of mental disorders, psychological distress, and mental health services use among lesbian, gay, and bisexual adults in the United States. J Consult Clin Psychol.

[9] Pega F, Smith L, Hamilton T (2012). Factors increasing and decreasing binge-drinking in young people attracted to more than one gender: a qualitative study of focus groups.

[10] Condit M, Kitaji K, Drabble L (2011). Sexual minority women and alcohol: intersections between drinking, relational contexts, stress and coping. J Gay Lesbian Soc Serv.

[11] Bos HM, Boschloo L, Schoevers RA (2015). Depression and anxiety in patients with and without same-sex attraction: differences in clinical expression, lifestyle factors, and vulnerability indicators. Brain Behav.

[12] Keyes KM, Hatzenbuehler ML, Hasin DS (2011). Stressful life experiences, alcohol consumption, and alcohol use disorders: the epidemiologic evidence for four main types of stressors. Psychopharmacology (Berl).

[13] Lea T, de Wit J, Reynolds R (2014). Minority stress in lesbian, gay, and bisexual young adults in Australia: associations with psychological distress, suicidality, and substance use. Arch Sex Behav.

[14] Hughes T, McCabe SE, Wilsnack SC (2010). Victimization and substance use disorders in a national sample of heterosexual and sexual minority women and men. Addiction.

[15] Badenes-Ribera L, Bonilla-Campos A, Frias-Navarro D (2016). Intimate partner violence in self-identified lesbians: a systematic review of its prevalence and correlates. *Trauma Violence Abuse*.

[16] Corliss HL, Grella CE, Mays VM (2006). Drug use, drug severity, and help-seeking behaviors of lesbian and bisexual women. J Women's Health (Larchmt).

[17] Steele LS, Daley A, Curling D (2017). LGBT identity, untreated depression, and unmet need for mental health services by sexual minority women and trans-identified people. *J Women's Health (Larchmt)*.

[18] Avery AM, Hellman RE, Sudderth LK (2001). Satisfaction with mental health services among sexual minorities with major mental illness. Am J Public Health.

[19] Senreich E (2009). A comparison of perceptions, reported abstinence, and completion rates of gay, lesbian, bisexual, and heterosexual clients in substance abuse treatment. J Gay Lesbian Ment Health.

[20] Jeong YM, Veldhuis CB, Aranda F (2016). Racial/ethnic differences in unmet needs for mental health and substance use treatment in a community-based sample of sexual minority women. J Clin Nurs.

[21] Garde EL, Manning V, Lubman DI (2017). Characteristics of clients currently accessing a national online alcohol and drug counselling service. Australas Psychiatry.

[22] McNair RP, Bush R (2016). Mental health help seeking patterns and associations among Australian same sex attracted women, trans and gender diverse people: a survey-based study. BMC Psychiatry.

[23] Stevens S (2012). Meeting the substance abuse treatment needs of lesbian, bisexual and transgender women: implications from research to practice. Subst Abuse Rehabil.

[24] Taliaferro JD, Lutz B, Moore AK (2014). Increasing cultural awareness and sensitivity: effective substance treatment in the adult lesbian population. J Hum Behav Soc Environ.

[25] McNair R, Hegarty K, Taft A (2015). Disclosure for same-sex attracted women enhancing the quality of the patient-doctor relationship in general practice. Aust Fam Physician.

[26] Hirsch O, Löltgen K, Becker A (2016). Lesbian womens’ access to healthcare, experiences with and expectations towards GPs in German primary care. *BMC Fam Pract*.

[27] Baldwin A, Dodge B, Schick V (2017). Health and identity-related interactions between lesbian, bisexual, queer and pansexual women and their healthcare providers. Cult Health Sex.

[28] Gelberg L, Andersen RM, Leake BD (2000). The behavioral model for vulnerable populations: application to medical care use and outcomes for homeless people. Health Serv Res.

[29] Stein JA, Andersen R, Gelberg L (2007). Applying the Gelberg-Andersen behavioral model for vulnerable populations to health services utilization in homeless women. J Health Psychol.

[30] Grella CE, Greenwell L, Mays VM (2009). Influence of gender, sexual orientation, and need on treatment utilization for substance use and mental disorders: findings from the California Quality of Life Survey. BMC Psychiatry.

[31] Sherbourne CD, Stewart AL (1991). The MOS social support survey. Soc Sci Med.

[32] Andresen EM, Malmgren JA, Carter WB (1994). Screening for depression in well older adults: evaluation of a short form of the CES-D (Center for Epidemiologic Studies Depression Scale). Am J Prev Med.

[33] Babor T, Higgins-Biddle J, Saunders JB (2001). The Alcohol Use Disorders Identification Test: guidelines for use in primary care.

[34] Nield J, Magnusson B, Brooks C (2015). Sexual discordance and sexual partnering among heterosexual women. Arch Sex Behav.

[35] Cruz TM (2014). Assessing access to care for transgender and gender nonconforming people: a consideration of diversity in combating discrimination. Soc Sci Med.

[36] Erlyana E, Fisher DG, Reynolds GL (2014). Medical service use among individuals receiving HIV prevention services in Los Angeles County. J Health Hum Serv Adm.

[37] Tam CW, Zwar N, Markham R (2013). Australian general practitioner perceptions of the detection and screening of at-risk drinking, and the role of the AUDIT-C: a qualitative study. BMC Fam Pract.

[38] Pennay A, Lubman DI, Frei M (2014). Alcohol: prevention, policy and primary care responses. Aust Fam Physician.

[39] Sherbourne CD (1988). The role of social support and life stress events in use of mental health services. Soc Sci Med.

[40] St Pierre M, Senn CY (2010). External barriers to help-seeking encountered by Canadian gay and lesbian victims of intimate partner abuse: an application of the barriers model. Violence Vict.

[41] O'Doherty L, Taket A, Valpied J (2016). Receiving care for intimate partner violence in primary care: barriers and enablers for women participating in the weave randomised controlled trial. Soc Sci Med.

[42] Lambrew JM, DeFriese GH, Carey TS (1996). The effects of having a regular doctor on access to primary care. Med Care.

[43] Andrews G, Henderson S, Hall W (2001). Prevalence, comorbidity, disability and service utilisation. Overview of the Australian National Mental Health Survey. Br J Psychiatry.

[44] McNair R, Szalacha LA, Hughes TL (2011). Health status, health service use, and satisfaction according to sexual identity of young Australian women. Womens Health Issues.

[45] Tjepkema M (2008). Health care use among gay, lesbian and bisexual Canadians. Health Rep.

[46] Steele LS, Tinmouth JM, Lu A (2006). Regular health care use by lesbians: a path analysis of predictive factors. Fam Pract.

[47] Bergeron S, Senn CY (2003). Health care utilization in a sample of Canadian lesbian women: predictors of risk and resilience. Women Health.

[48] van Dam MAA, Koh AS, Dibble SL (2001). Lesbian disclosure to health care providers and delay of care. J Gay Lesbian Med Assoc.

[49] Diamant AL, Wold C, Spritzer K (2000). Health behaviors, health status, and access to and use of health care: a population-based study of lesbian, bisexual, and heterosexual women. *Arch Fam Med*.

[50] Penn PE, Brooke D, Mosher CM (2013). LGBTQ persons with co-occurring conditions: perspectives on treatment. Alcohol Treat Q.

